# Supplementary material for: Electropolymerization of robust conjugated microporous polymer membranes for rapid solvent transport and narrow molecular sieving
Source: Nat Commun. 2020 Oct 21;11:5323. doi: 10.1038/s41467-020-19182-1 (PMC7578036; doi:10.1038/s41467-020-19182-1)
Supplement: Supplementary file 1 — Supplementary Information [file 41467_2020_19182_MOESM1_ESM.docx]

Supplementary Information

Electropolymerization of Robust Conjugated Microporous Polymer Membranes for Rapid Solvent Transport and Narrow Molecular Sieving

Zongyao Zhou ^1^, Xiang Li ^1^, Dong Guo ^1^, Digambar B. Shinde ^1^, Dongwei Lu ^1^, Long Chen ^2^, Xiaowei Liu ^1^, Li Cao ^1^, Ammar M. Aboalsaud ^1^, Yunxia Hu ^3^, Zhiping Lai ^1,^*

1 Advanced Membranes and Porous Materials Center, Division of Physical Science and Engineering, King Abdullah University of Science and Technology (KAUST), Thuwal, 23955-6900, Saudi Arabia

2 Core Lab, King Abdullah University of Science and Technology (KAUST), Thuwal, 23955-6900, Saudi Arabia

3 State Key Laboratory of Separation Membranes and Membrane Processes, School of Materials Science and Engineering, Tiangong University, Tianjin, 300387, P.R. China

* Corresponding author E-mail: Zhiping.lai@kaust.edu.sa

# Simulation of the molecular geometry of the SpCz monomer

The molecular geometry of the SpCz monomer is simulated by the First-principles DFT simulation using the B3LYP function of Gaussian 09.^1-3^ The 6-311g++ basis functions were applied to the systems.^4^

# The Young’s modulus measurements by peak force quantitative nanomechanical mapping (PFQNM)

The measurement was conducted on a Dimension Icon Atomic Force Microscope system (Bruker, Santa Barbara, CA) equipped with a Stargate scanner (maximum scan size: 90 μm) and a silicon probe (RTESPA-300, Bruker). The scanning rate was set to 0.5 Hz. The spring constant of the cantilever of the probe was calibrated by the Sader method and gave a value of 28.475 N/m in our measurements. The PFQNM mode is based on peak force tapping that applies a very fast force curve at every pixel by modulating the Z piezo at ~1 kHz with an amplitude of 30 nm. The peak force of each of these curves, which was set the value around 20 nN, is then used as the imaging feedback signal. The Hertzian (Spherical) model is employed to analyze the modulus of the film in the measurements. The Young’s Modulus E of the film is calculated by equation (1),

$F=\frac{4}{3}\frac{E}{(1-v^{2})}R^{1/2}h^{3/2}$ (1)

where *F* is the applied load, *ν* is the Poisson’s ratio of the sample, *R* is the tip radius, and *h* is the indentation depth. Analysis of the force curve data is done in real-time, providing a map of multiple mechanical properties. This mode controls the tip/sample interaction directly to reduce the deformation of the sample and the contact area. Therefore, damages to the probe or sample are minimized, and a higher resolution is achieved.

# Supplementary Figures


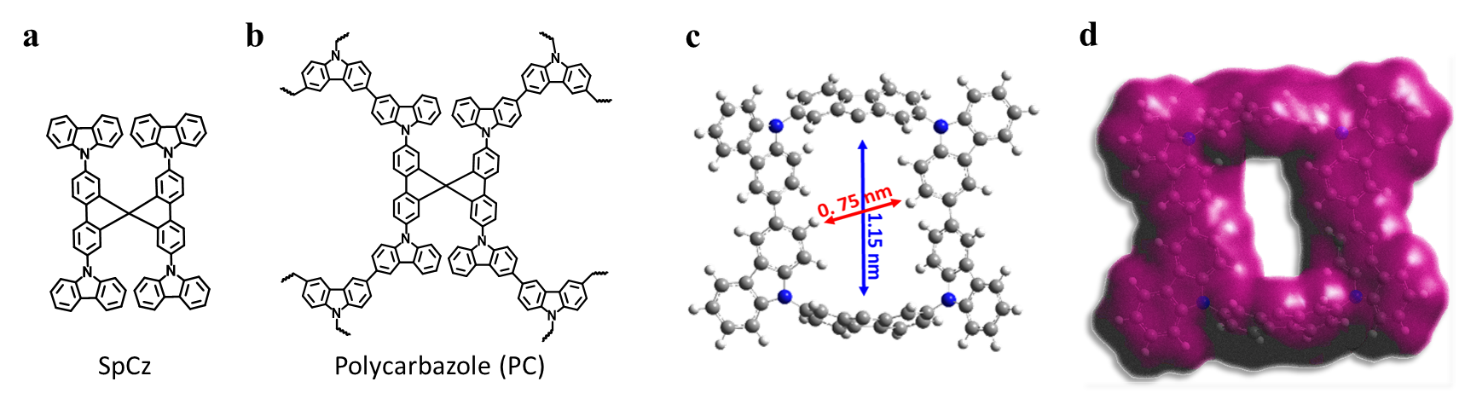


**Fig. S1** **a** Molecular structure of monomer SpCz. **b** Formation of polycarbazole through the linkages along with the four carbazole groups of SpCz. **c** Geometric optimization of the SpCz monomer showing an intrinsic space with dimension 1.15 nm by 0.75 nm within the molecule. **d** The molecular electrostatic potential map of SpCz monomer.


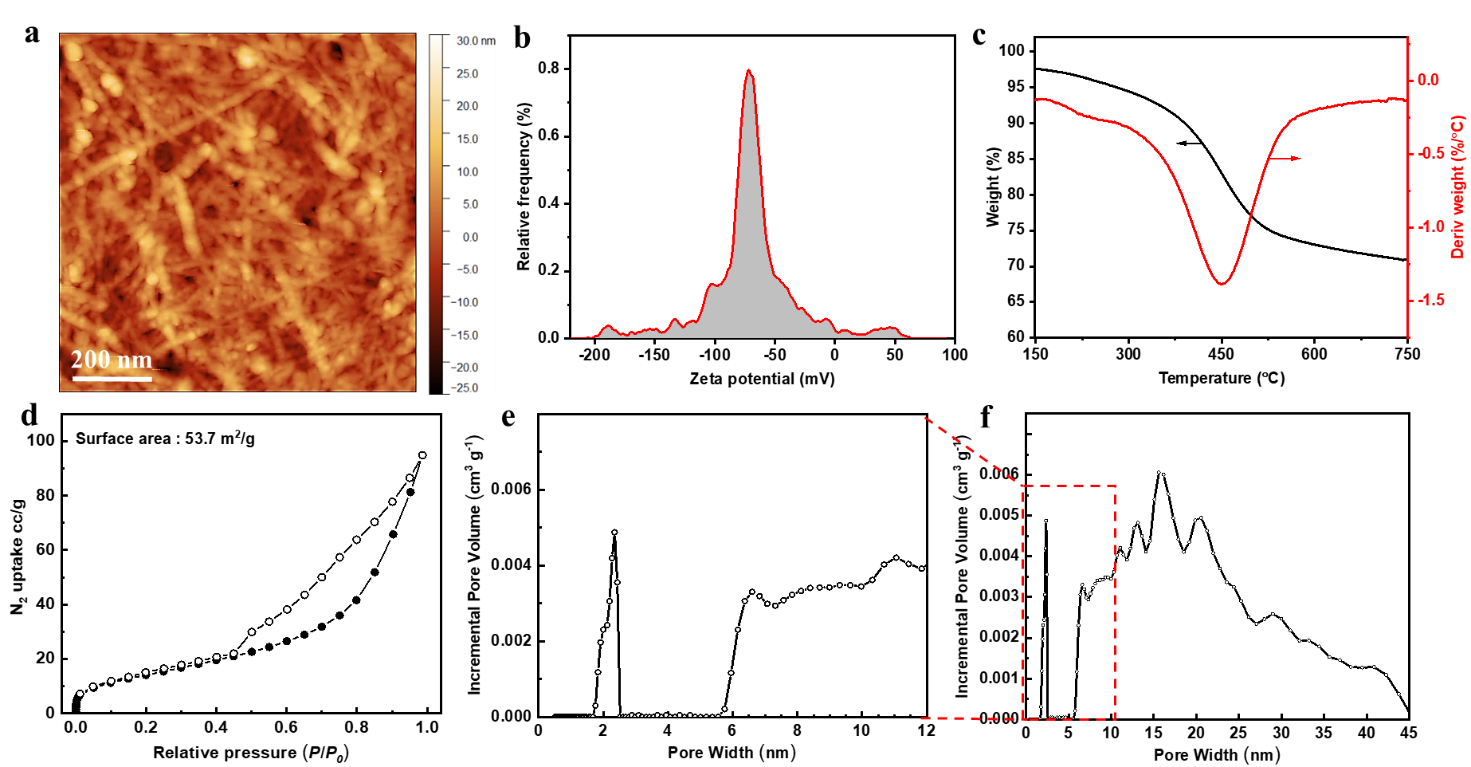


**Fig. S2** Characterization of the PDA-CNT porous support. **a** AFM image. **b** Zeta potential of the PDA-CNT suspension. **c** Thermogravimetric analysis. **d** N_2_ adsorption isotherm. **e** A magnified pore size distribution of **f** showing the sharp peak around 2.2 nm attributed to the CNT internal channels. **f** Pore size distribution. The pore size distribution is calculated from the adsorption isotherm using the NLDFT method.


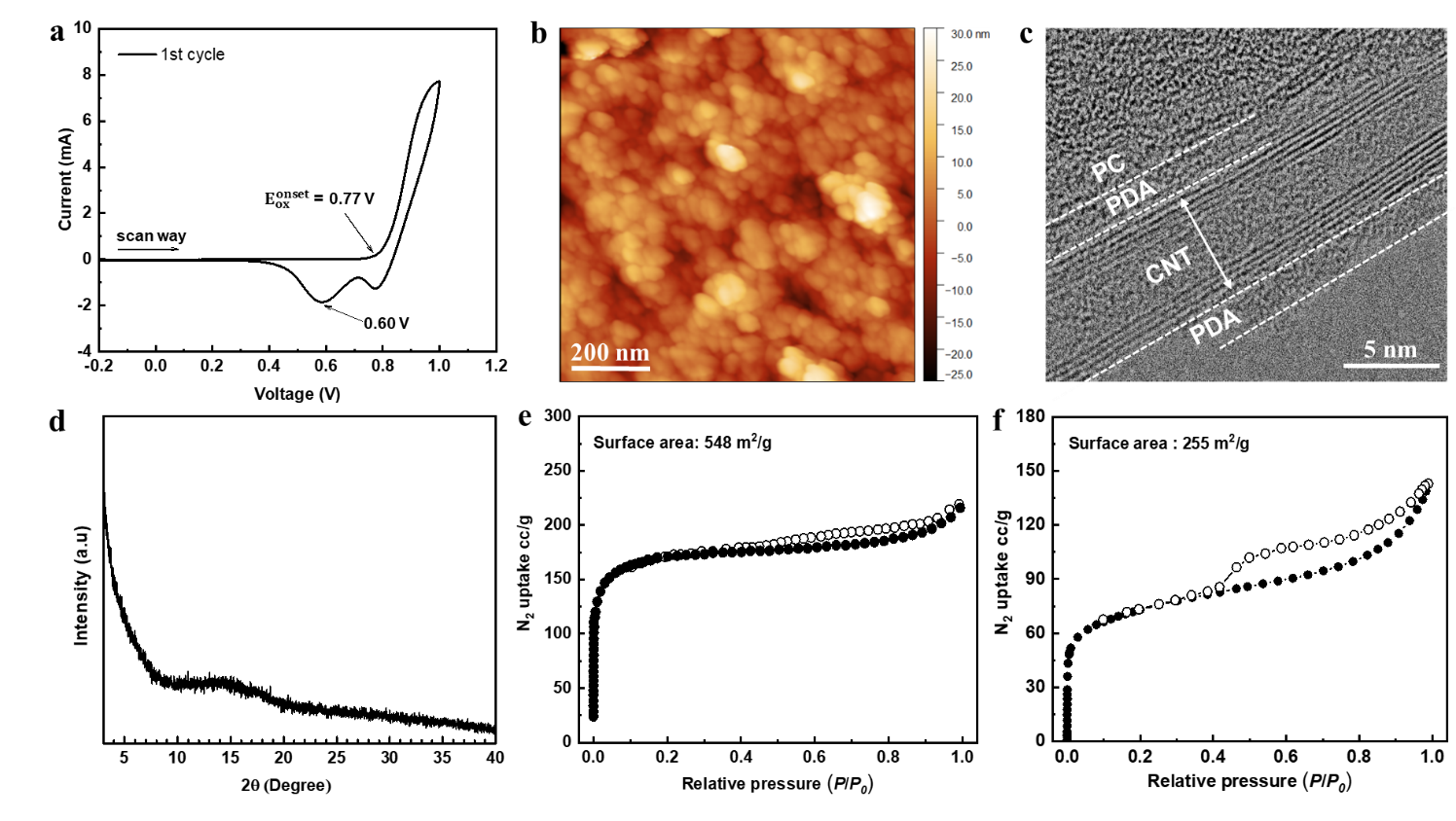


**Fig. S3** Characterization of the CNT-EP-PC15 membrane. **a** CV profile of the EP process recorded for the first scan cycle. **b** AFM image. **c** TEM image in the intermedia layer. **d** XRD pattern. **e** N_2_ adsorption isotherm of the top layer. **f** N_2_ adsorption isotherm of the intermediate and central regions.


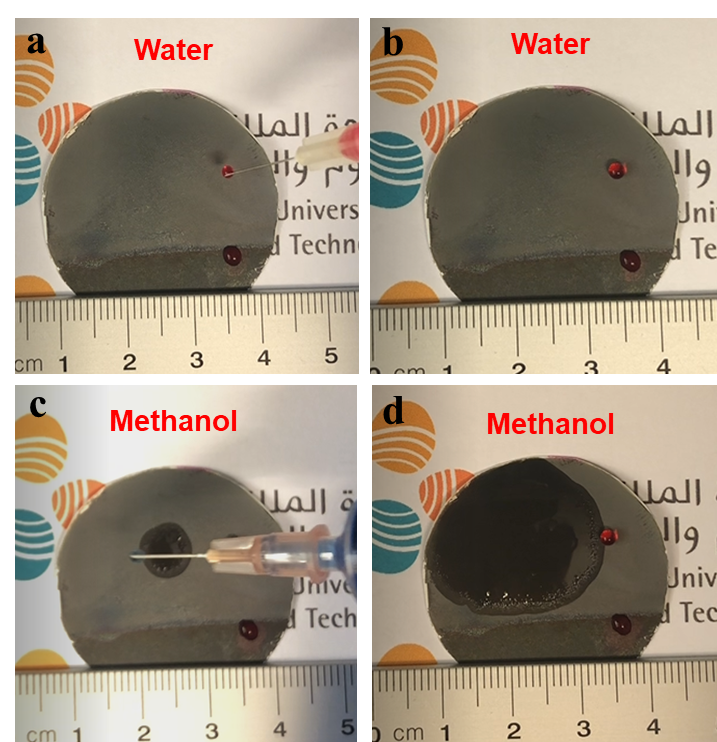


**Fig. S4** Wettability of the CNT-EP-PC15 membrane towards water and methanol. **a**, **b** Photographs of water droplets placed on the membrane. **c**, **d** Photographs of methanol droplets on the membrane.

| **a** | **b** | **c** |
| --- | --- | --- |
| 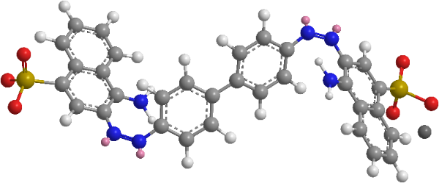 | 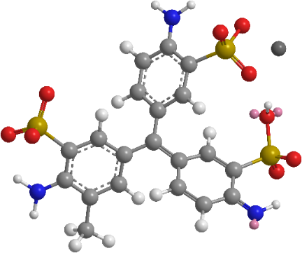 | 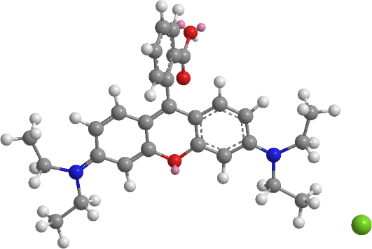 |
| Congo Red  MW: 697  Size: 1.3 nm × 0.9 nm | Acid Fuchsin  MW: 586  Size: 1.4 nm × 1.2 nm | Rhodamine B  MW: 479  Size: 1.7 nm × 1.3 nm |
|  | | |
| **d** | **e** | **f** |
| 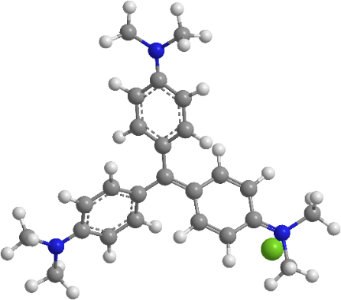 | 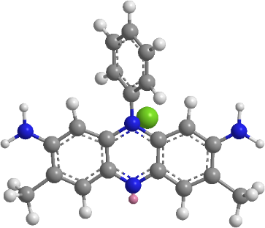 | 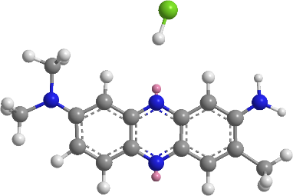 |
| Crystal Violet  MW: 408  Size: 1.4 nm × 1.3 nm | Safranine O  MW: 351  Size: 1.1 nm × 1.1 nm | Neutral Red  MW: 289  Size: 1.2 nm × 0.8 nm |

**Fig. S5** Molecular structures of the studied dyes. **a**, **b** and **c** are cationic dyes. **d** and **e** are anionic dyes. **f** is a neutral dye. The molecular structures and sizes are simulated and estimated using the Molecular Mechanics 2 method in Chem3D.


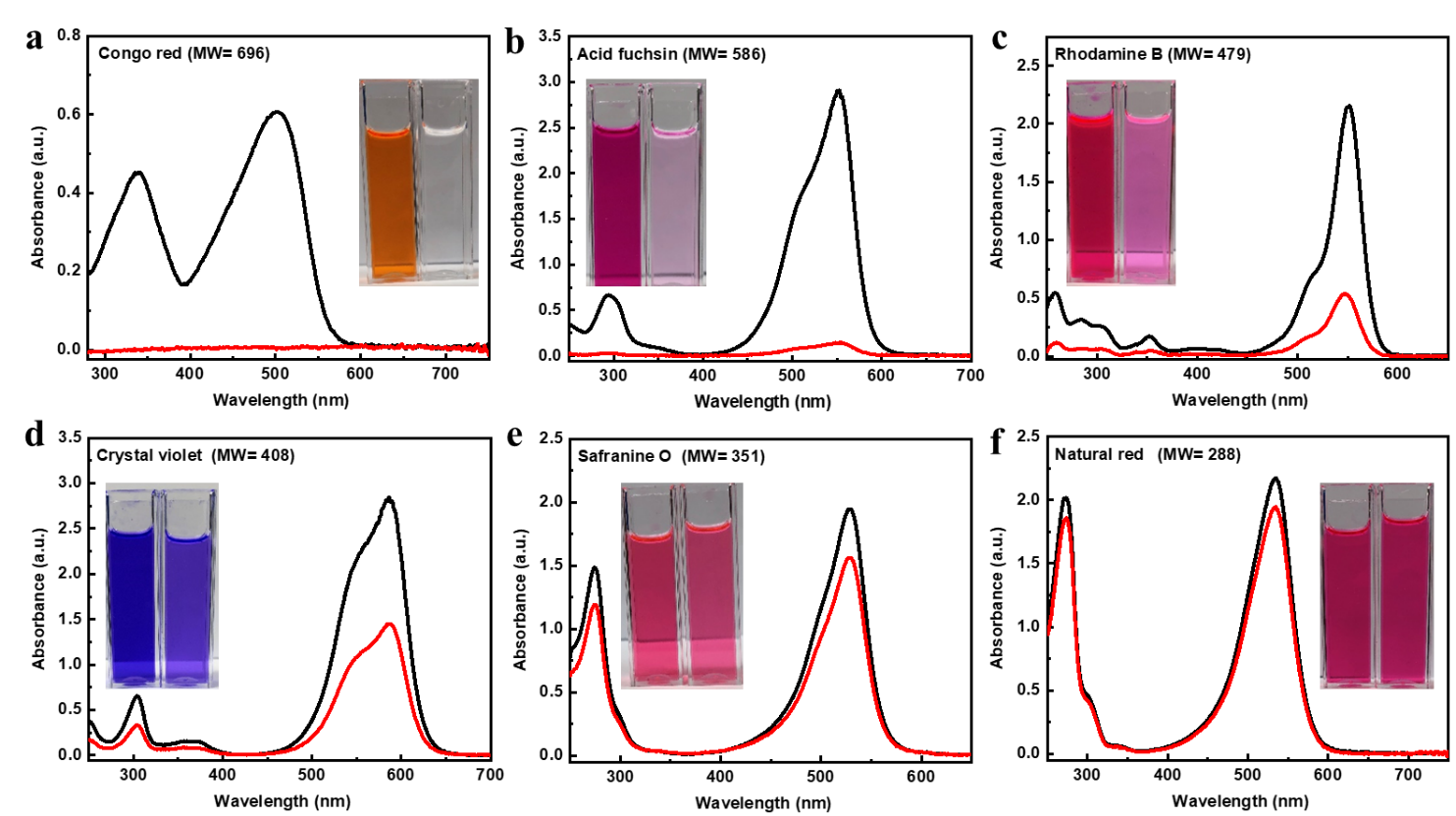


**Fig. S6** UV–vis absorbance spectra of the feed (black curve) and permeate (red curve) solutions measured in the permeation studies of dyes on the CNT-EP-PC15 membrane under the transmembrane pressure drop of 1 bar.

**Fig. S7** Long-term filtration tests of various organic solvents on the CNT-EP-PC15 membrane under the transmembrane pressure drop of 1 bar.

# Supplementary Tables

**Table S1.** Comparison of mechanical strength of the CNT-EP-PC15 membrane with some common OSN polymer membranes.

| Membranes | Strain (%) | Tensile Strength (MPa) | Reference |
| --- | --- | --- | --- |
| PSF | —— | 3.33 | 5 |
| CA | 11.2 | 39 | 6 |
| PVDF | 33.7 | 7.5 | 6 |
| PAN | 4.9 | 16.5 | 6 |
| PES | ~45 | ~10 | 7 |
| CNT-EP-PC15 | 18.9 | 26.5 | This work |

# Reference

1. Frisch MJ, Trucks GW, Schlegel HB, et al., Gaussian 09, Revision B.01, Gaussian Inc., Wallingford CT (2010).

2. Lee C, Yang W, Parr RG. Development of the Colle-Salvetti correlation-energy formula into a functional of the electron density. *Phys Rev B* **37**, 785-789 (1988).

3. Becke AD. Density-functional exchange-energy approximation with correct asymptotic behavior. *Phys Rev A* **38**, 3098-3100 (1988).

4. Petersson GA, Al‐Laham MA. A complete basis set model chemistry. II. Open‐shell systems and the total energies of the first‐row atoms. *J Chem Phys* **94**, 6081-6090 (1991).

5. Ammar A, Al-Enizi AM, AlMaadeed MA, Karim A. Influence of graphene oxide on mechanical, morphological, barrier, and electrical properties of polymer membranes. *Arabian J Chem* **9**, 274-286 (2016).

6. Liang H-Q, Wu Q-Y, Wan L-S, Huang X-J, Xu Z-K. Polar polymer membranes via thermally induced phase separation using a universal crystallizable diluent. *J Membr Sci* **446**, 482-491 (2013).

7. Elele E, Shen Y, Tang J, Lei Q, Khusid B, Tkacik G, Carbrello C. Mechanical properties of polymeric microfiltration membranes. *J Membr Sci* **591**, 117351 (2019).
