# Supplementary material for: Electropolymerization of robust conjugated microporous polymer membranes for rapid solvent transport and narrow molecular sieving
Source: Nat Commun. 2020 Oct 21;11:5323. doi: 10.1038/s41467-020-19182-1 (PMC7578036; doi:10.1038/s41467-020-19182-1)
Supplement: Supplementary file 3 — Description of Additional Supplementary Files [file 41467_2020_19182_MOESM3_ESM.pdf]

Description of Additional Supplementary Files

File Name: Supplementary Movie 1
Description: Movie to show a CNT-EP-PC membrane holding substantial weight without break

File Name: Supplementary Movie 2
Description: Movie to show a CNT-EP-PC membrane bearing substantial bending without break.

File Name: Supplementary Movie 3
Description: Movie to show the dual super-hydrophobicity and super-oleophilicity of the CNT-EP-PC membrane
